# *Catalpa lutea* (Bignoniaceae), a new species from north, central, and east China

**DOI:** 10.3897/phytokeys.270.171460

**Published:** 2026-01-28

**Authors:** Ying Liu, Zhen-Yu Li, Jiang-Tao Zhang, Wan-Ting Ge, Ming-Gang Zhang, Nan Lu, Wen-Jun Ma, Jun-Hui Wang

**Affiliations:** 1 State Key Laboratory of Tree Genetics and Breeding, Key Laboratory of Tree Breeding and Cultivation of State Forestry Administration, Research Institute of Forestry, Chinese Academy of Forestry, Beijing 100091, China Chinese Academy of Forestry and Northeast Forestry University Beijing China; 2 State Key Laboratory of Tree Genetics and Breeding, Chinese Academy of Forestry and Northeast Forestry University, Beijing 100091, China Research Institute of Forestry, Chinese Academy of Forestry Beijing China; 3 State Key Laboratory of Systematic and Evolutionary Botany, Institute of Botany, Chinese Academy of Sciences, Beijing 100093, China Institute of Botany, Chinese Academy of Sciences Beijing China; 4 Henan Academy of Forestry, Zhengzhou 450008, China Henan Academy of Forestry Zhengzhou China; 5 Guizhou Academy of Forestry, Guiyang 550005, China Guizhou Academy of Forestry Guiyang China

**Keywords:** Bignoniaceae, *
Catalpa
*, chloroplast genome, morphology, SNPs

## Abstract

*Catalpa lutea* J.H.Wang, W.J.Ma & Z.Y.Li, a new species of Bignoniaceae from north, central, and east China, is described and illustrated. By combining recent morphological comparisons with phylogenetic analyses of nuclear single-nucleotide polymorphisms (SNPs) and the chloroplast genome, we demonstrate that *C. lutea* is readily distinguishable from its congeners, including *Catalpa fargesii* Bureau and other Chinese species of section *Catalpa*. A detailed morphological description, illustrations, and molecular data are provided. While conforming to sect. *Catalpa* in its typically ovate leaf blades, didynamous stamens, and five corolla lobes, *C. lutea* is unequivocally distinguished by its narrowly ovate crown, yellow heartwood, bright yellow anthers, filaments with purple dots, conspicuously thickened deep purple striations within the corolla tube, and floral spots exceeding 1 mm in diameter. Additional diagnostic characters include young foliage frequently flushed purple, mature leaves green with a purple tinge on occasion, and 3–4 nectaries at the leaf axils on the abaxial surface. The conservation status of this species is assessed as “Data Deficient” (DD) according to the IUCN Red List Categories and Criteria.

## Introduction

Bignoniaceae Juss. belongs to Lamiales (Olmstead et al. 1993; Angiosperm Phylogeny Group II 2003) and includes about 110 genera and more than 800 species (http://www.britannica.com/plant/Bignoniaceae). Several genera include species of horticultural importance in tropical and temperate regions, including *Bignonia*, *Campsis*, *Catalpa*, *Jacaranda*, *Spathodea*, and *Tabebuia* (Olmstead et al. 2009). The genus *Catalpa* Scop. (Bignoniaceae, tribe Catalpeae DC. ex Meisn.) contains ten natural species distributed in two sections, *C.* sect. *Catalpa* and *C.* sect. *Macrocatalpa* Griseb. Section *Macrocatalpa* contains four natural species, which are only found in the Greater Antilles (Fischer et al. 2004; Olsen and Kirkbride 2017). Section *Catalpa* includes six natural species and two hybrids, which are distributed in China and North America (Li 1952; Wen 1999; Li 2008; Yih 2012; Li et al. 2022).

All species of sect. *Catalpa* are deciduous trees occurring in temperate to subtropical regions. The species are often described as among the most beautiful of the hardy flowering trees, with thyrses of white or yellow flowers or racemes or corymbs of pink flowers (Meyer 1907; Dirr 2009; Grimshaw and Olsen 2011). *C. bignonioides* Walter and *C. speciosa* Teas are endemic to North America and show paniculate inflorescences, a white corolla, a circular corolla orifice, and relatively wide capsules (0.9–1.7 cm). The Chinese endemic species *C. ovata* G. Don is similar to the North American species in its paniculate inflorescences and circular corolla orifice, but *C. ovata* exhibits a small, yellow corolla and narrower capsules (0.4–0.7 cm). The three other species endemic to China (*C. bungei* C.A. Mey, *C. duclouxii* Dode, and *C. fargesii* Bureau; Li 2008) present corymbose, racemose, or paniculate inflorescences, a transversely elliptic corolla orifice, and relatively narrow capsules (0.3–0.6 cm width), but there are differences in the color of the wood and young branches, indumentum type, color and shape of flowers, and distribution of these species. Additionally, *C. tibetica* Forrest has been recognized as a synonym of *C. bignonioides* (Kirkbride and Olsen 2011).

**Figure 1.**
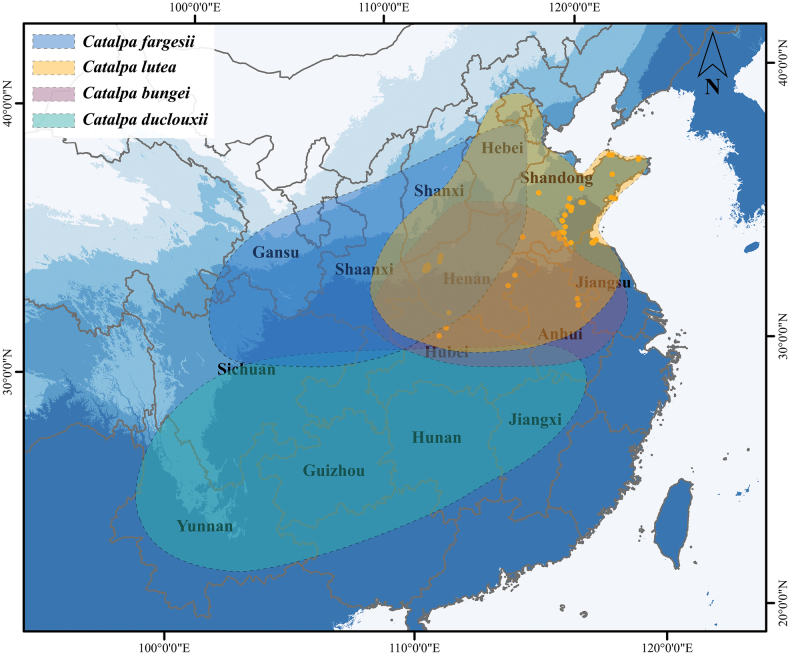
Distribution of the *Catalpa* species in China. The blue background on the map indicates rainfall in the region, with darker blue representing more abundant precipitation. The orange spots indicate the localities where wild specimens of *Catalpa lutea* were collected. The orange area denotes the main distribution range of *C. lutea*, while the other colors represent the principal distribution areas of *C. bungei*, *C. duclouxii*, and *C. fargesii*, respectively. Map approval number: GS20240650.

To assess the current status of wild Chinese *Catalpa* resources and to provide a foundation for biodiversity conservation and sustainable utilization, we initiated nationwide field surveys in 2009 and have since assembled an extensive germplasm collection. In 2010, an unfamiliar wild population specimen was first collected in Luanchuan County, Henan Province. Detailed comparison with the protologues presented in “Flora of China” (Zhang and Santisuk 1998) revealed consistent morphological divergence from all previously recognized members of sect. *Catalpa*. Over the subsequent 15 years, extensive wild populations were documented across northwestern Shaanxi Province, eastern Anhui and Shandong Provinces, and coastal Lianyungang City, Jiangsu Province, including veteran trees exceeding 200 years of age (Fig. 1). Consistent with Olsen and Kirkbride (2017), this taxon maintains both distributional and morphological differentiation from congeners. Historical records indicate that this yellow-wooded lineage has been taxonomically distinguished from other *Catalpa* species since as early as 1500 years ago. Integrated morphological and molecular phylogenetic analyses confirm that this lineage represents a previously unrecognized natural species of *Catalpa*. Herein, we formally describe it as *Catalpa lutea* J.H.Wang, W.J.Ma & Z.Y.Li, sp. nov. and discuss its systematic relationships.

## Material and methods

### Morphological investigation

Morphological observations and measurements of the new species were carried out on both living plants in the field and dried specimens from herbaria (**HITBCI**, **PE**, **CAF**, **YCP**, and **RAF**). Photographs were taken in the field. All morphological characteristics were studied under a dissecting microscope and described using the terminology presented by Olsen and Kirkbride (2017).

### Sample collection, DNA sequencing, and chloroplast genome assembly

To construct a phylogenetic tree of the genus *Catalpa*, we selected the monotypic sister genus *Chilopsis* D. Don as the outgroup, represented by one sample of *Chilopsis linearis*. A total of 29 *Catalpa* samples were included, covering seven species of sect. *Catalpa* (Suppl. material 1). DNA was extracted from silica gel-dried leaves using the DNAquick Plant System (Tiangen Biotech Co., Ltd., Beijing, China). DNA quality and concentration were assessed by 1% agarose gel electrophoresis, a Nanodrop 2000 spectrophotometer (Thermo Fisher Scientific Inc., Waltham, MA, USA), and a QubitTM 2.0 Fluorometer (Thermo Fisher Scientific Inc., Waltham, MA, USA). Sequencing libraries were prepared with the NEBNext Ultra DNA Library Prep Kit (New England Biolabs, Ipswich, MA, USA). The DNA samples were indexed by tags and then sequenced on a DNBSEQ T7 platform at Glbizzia Biosciences Co., Ltd., Beijing, China. Single nucleotide polymorphisms (SNPs) were identified by aligning whole-genome resequencing reads to the *Catalpa bungei* reference genome. High-quality reads were mapped using BWA v0.7.17 (Li and Durbin 2009), and duplicate reads were removed with SAMTOOLS (Li et al. 2009). SNP calling was performed using GATK v4.4.0.0, with filtering based on the following thresholds: QD < 2.0, MQ < 20.0, FS > 60.0, MQRankSum < –12.5, and ReadPosRankSum < –8.0. Additional filtering was conducted with VCFtools v0.1.16 (Danecek et al. 2011) to exclude SNPs with >20% missing data and a minor allele frequency < 0.05. In parallel, chloroplast genomes of all 30 samples were assembled separately using GetOrganelle v1.7.5 (Jin et al. 2020), based on the raw sequencing data and reference chloroplast genomes of six *Catalpa* species from Li et al. (2022). The assembled chloroplast genomes were annotated and visualized with CPGAVAS2.

### Phylogenetic analyses

Phylogenetic analyses were conducted using both maximum likelihood (ML) and Bayesian inference (BI). For ML analysis, RAxML v1.2.1 (Stamatakis 2014) was used with the best-fit model selected by jModelTest2 (Darriba et al. 2012). Bootstrap support was evaluated with 1,000 replicates. Separate ML trees were constructed for nuclear SNPs and chloroplast genomes, with *Chilopsis linearis* set as the outgroup. For BI analysis, sequences were aligned using MAFFT (Nakamura et al. 2018) and converted to NEXUS format. XML input files were prepared with BEAUti v1.8.2 (Drummond et al. 2012). Markov chain Monte Carlo (MCMC) analyses were run for 10 million generations in BEAST v1.8.2 (Drummond et al. 2012), with sampling every 1,000 generations starting from a random tree. Convergence was assessed using Tracer v1.6, ensuring effective sample size (ESS) values > 200 for all parameters. Phylogenetic trees were visualized using FigTree v1.4.4 and Adobe Illustrator 2021.

## Results

### Phylogenetic relationships

**Figure 2.**
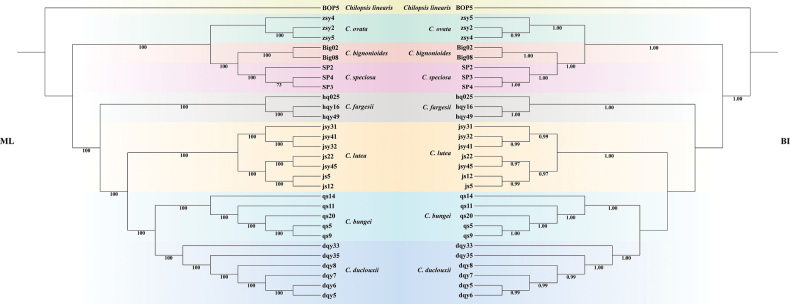
Phylogenetic relationships of *Catalpa* inferred from nuclear SNP data of 30 samples using maximum likelihood (ML) and Bayesian inference (BI). The bootstrap support values (BS) and posterior probabilities (PP) are listed at each node.

After data filtering, we obtained 1,427,052 nuclear SNP sites (available at https://ngdc.cncb.ac.cn/gsa, accession number: CRA034837). The phylogenetic tree reconstructed from these SNPs is presented in Fig. 2. The topologies derived from ML and BI analyses were largely congruent. Most nodes in both the ML and BI trees received full support (BS = 100%, PP > 0.97), except for a small number of nodes. The phylogeny based on the SNP dataset showed that samples of each taxon in sect. *Catalpa* formed monophyletic clades. The species were divided into two major clades: *C. ovata* clustered with the North American species *C. bignonioides* and *C. speciosa* in one clade, while the remaining *Catalpa* species distributed in China formed the other clade. Within the latter clade, the new species was positioned close to the clade comprising *C. bungei* and *C. duclouxii* (PP = 1.00, BS = 100%).

**Figure 3.**
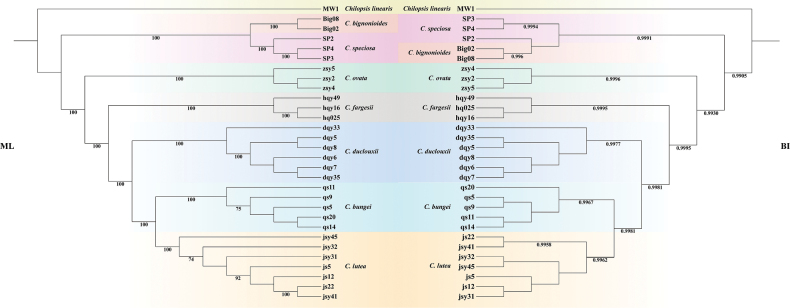
Phylogenetic relationships of *Catalpa* inferred from 30 complete chloroplast genomes using maximum likelihood (ML) and Bayesian inference (BI). The bootstrap support values (BS) and posterior probabilities (PP) are listed at each node.

The chloroplast genomes of the seven species from sect. *Catalpa* exhibited similar structural characteristics (chloroplast genome data are available at https://ngdc.cncb.ac.cn/genbase/review/97cdb7928376, accession numbers: C_AA133069.1–C_AA133098.1). Their lengths ranged from 157,890 bp (*C. fargesii*) to 158,355 bp (*C. ovata*). The phylogenetic tree reconstructed from the complete chloroplast genomes is presented in Fig. 3. Consistent with the SNP tree, samples of each taxon formed monophyletic clades in the plastid phylogeny. However, unlike the SNP tree, the chloroplast genome tree revealed that the North American species *C. bignonioides* and *C. speciosa* constituted one clade, while *C. ovata* clustered with the other Chinese taxa in another major clade. The new species formed a sister clade to *C. bungei* with strong support (PP = 0.9962, BS = 100%).

### Taxonomic treatment

#### Catalpa lutea

Taxon classificationPlantaeLamialesBignoniaceae

J.H.Wang, W.J.Ma & Z.Y.Li sp. nov.

B0700963-9B6C-5AA1-ABFD-20A0846E63FE

urn:lsid:ipni.org:names:77375729-1

**Figure 4.**
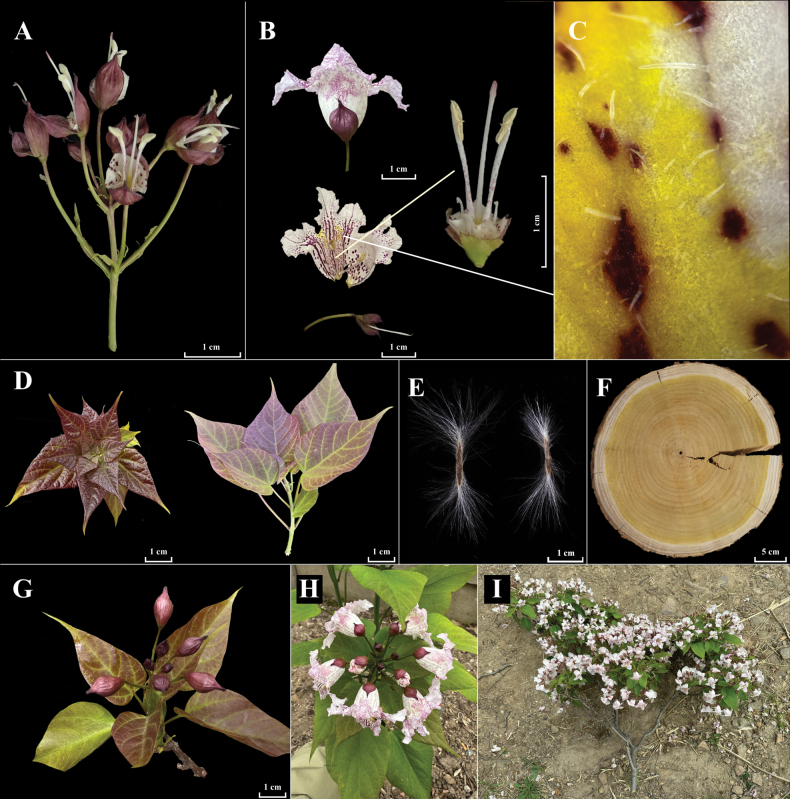
*C. lutea*. **A**. Inflorescence; **B**. Flower, pistil, and stamen; **C**. Spots and indumentum within the corolla throat; **D**. Young blade; **E**. Seed; **F**. Wood cross-section; **G, H**. Inflorescences at different developmental stages; **I**. Flowering branch. Photographs taken by Ying Liu.

**Figure 5.**
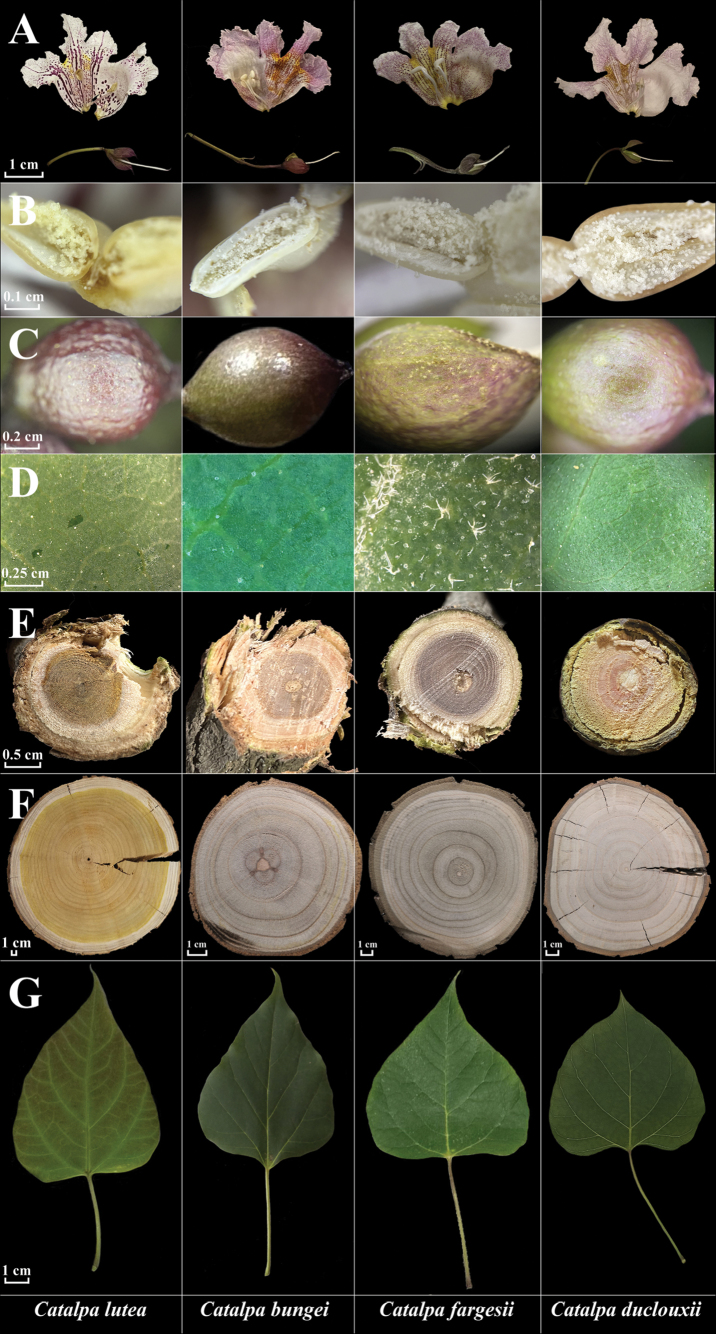
*C. lutea* and related species. Column 1, *C. lutea*; column 2, *C. bungei*; column 3, *C. fargesii*; column 4, *C. duclouxii*. **A**. Flower morphology; **B**. Anther; **C**. Flower bud and calyx; **D**. Blade indumentum; **E**. Cross-section of branches four or more years old; **F**. Heartwood color; **G**. Blade. Photographs taken by Ying Liu and Wen-Jun Ma.

**Figure 6.**
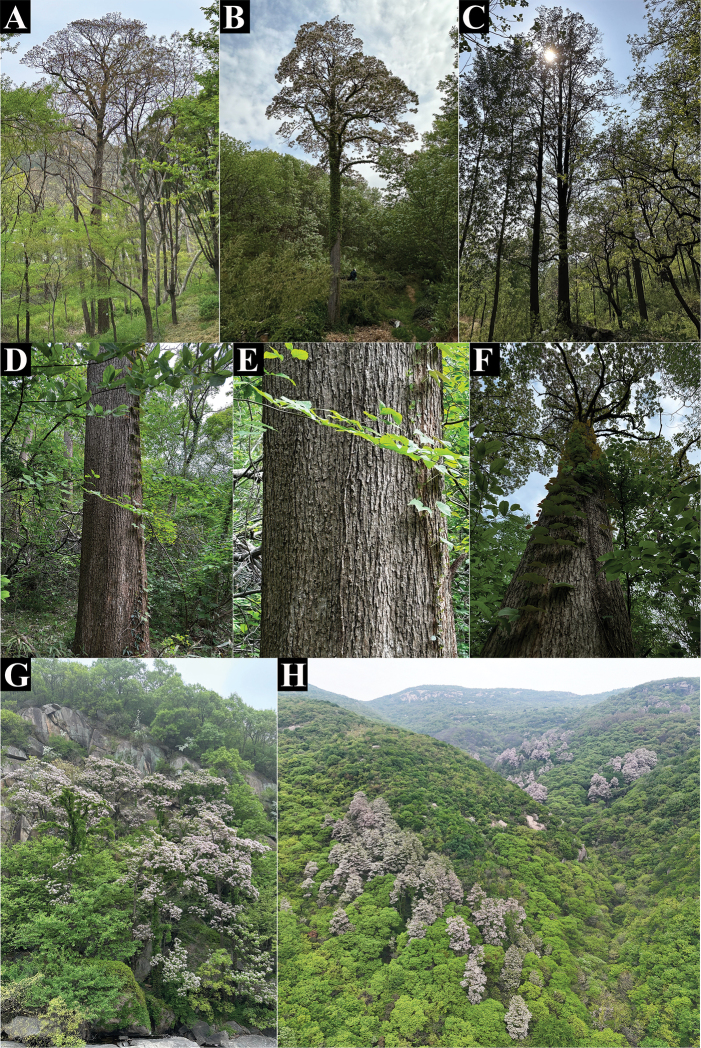
**A–C**. Mature trees of *C. lutea*; **D–F**. Habitat and bark of a 200+ year-old ancient *C. lutea* individual; **G, H**. Habitat.

Figs 4, 5, 6 Chinese name: 金丝楸

##### Type.

China • Henan Province, Luanchuan County, Qiuba Township, 34°07'59.6"N, 111°33'30.88"E, 115 m a.s.l., 26 Aug. 2019, *W.J. Ma* CATSQ002 (holotype: CAF!, barcode no. 10023403; isotypes: PE!, CAF!).

##### Diagnosis.

*C. lutea* is easily distinguished from all other species of *Catalpa* by the following combination of characters: narrowly ovate crown; yellow heartwood; yellow anthers; yellow xylem appearing in four-year-old branches and older; filaments with scattered purple spots and a purplish stigma; darker, thicker, and more prominent purple striations inside the corolla tube, with spots exceeding 1 mm in diameter; gray-brown bark that is neither warped nor exfoliating; leaf blades markedly longer than wide; young foliage frequently flushed purple, mature leaves green with occasional purple tinge; leaves of sprouting shoots suborbicular and 3–5-lobed; abaxial surface bearing 3–4 nectaries at the junctions of the veins on the lower leaf surface, two nectaries at the base. 2n = 40.

*C. lutea* is morphologically similar to *C. bungei* in its slender petioles, corymbose inflorescences, and white corolla with many purple spots. However, *C. lutea* differs from *C. bungei* by having a transversely elliptic corolla orifice, thicker petals, larger corolla spots, significantly fewer capsules, and a flowering phenology that is about 8 days earlier in regions of sympatry, in addition to its distribution extending to eastern coastal China. Phylogenetic analysis based on genome-wide SNP data revealed that *C. lutea* was sister to a clade comprising *C. duclouxii* and *C. bungei*, with strong statistical support. Phylogenetic analysis based on the complete chloroplast genome indicated that *C. lutea* formed a sister clade to *C. bungei*. A detailed morphological comparison of the species most similar to *C. lutea* is provided in Table 1.

**Table 1.** Morphological comparison of *Catalpa lutea* and its most morphologically similar related species.

Character	*Catalpa lutea*	*Catalpa bungei*	*Catalpa fargesii*	*Catalpa duclouxii*
Wood	sapwood white, heartwood yellow, annual rings indistinctly brownish	sapwood gray-white, heartwood gray-brownish, annual rings brownish	sapwood gray-white, heartwood gray-brownish, annual rings brownish	sapwood gray-white, heartwood gray-brown, annual rings brown
Indumentum	Generally glabrous, occasionally with sparse simple hairs	glabrous	simple, stellate, and dendroid hairs	glabrous
Young shoots	brown green to brown gray, scattered narrowly elliptic to elliptic lenticels	yellow-brown to brown, densely with orbiculate to elliptic lenticels	gray to brown, densely with orbiculate to elliptic lenticels	light gray, densely with orbiculate to broadly lenticels
Leaf blades	homomorphic, longer than wide or about as long as wide, purple green to green	homomorphic, longer than wide, green to deep green	homomorphic, longer than wide, deep green	homomorphic, longer than wide, deep green
Inflorescence	racemose-corymbose, rarely partly branched, 3–13-flowered	racemose to corymbose, not branched, 6–18-flowered	racemose to corymbose, 3–12-flowered	racemose to paniculate, (12–)15–38-flowered or more
Corolla	with scattered large deep purple spots, 9–12 rows of thick deep purple stripes, slightly raised 2-longitudinal ridges	with densely fog-like purplish pink microspots, 9–21 fine, discontinuous purplish-pink stripes and slightly raised 2-longitudinal ridges	with densely fog-like purplish pink microspots and 14–23 fine, discontinuous purplish-pink stripes and obviously raised 2-longitudinal ridges	with densely fog-like purplish pink microspots and 16–36 fine, discontinuous purplish-pink stripes and strongly raised 2-longitudinal ridges
Anthers	yellow	white	white	white
Filaments	white, scattered purple dots	white, without dots	white, without dots	white, without dots
Infructescence	with 1–2 capsules	with 1–3 capsules	with 1–3 capsules	with (2)3–10 capsules or more
Crown shape	crown ovate-conic to broadly ovoid	Ovate to broadly ovate	broadly ovate to umbrella-shaped	Narrowly ovate to ovate

##### Description.

Deciduous trees, 3–30 m tall, 30–100+ cm DBH, crown ovate-conic to broadly ovoid. **Wood**: with dark gray-brown sapwood and yellow heartwood. Young trunks with brown gray, smooth bark and scattered grayish lenticels; old trunks dark gray, vertically furrowed, and transversely fissured; young shoots brown green to brown gray, scattered narrowly elliptic or elliptic lenticels, mostly glabrous, occasionally sparsely hispidulous, glabrous when older. **Bark**: irregularly cracked; bark lobes horizontally broken into rectangular lobes and not warped, thick, and persistent. Leaves opposite or in whorls of three, unequal in size in each pair or each whorl, sparsely hispidulous when young, glabrate when older. **Petioles**: 3–9 cm long, 1–3 mm wide, pale green to red-purple; **leaf blades**: chartaceous, red-brownish when young, turning deep green with age, deltoid-ovate to broadly ovate, 7–14 × 5–12 cm, distinctly longer than the width, truncate or shallowly cordate at base, margin entire, apex cuspidate or caudate; mid-rib with 3–4 arcuate secondary veins on each side, 3 palmate-nerved at base, abaxial veins prominent; abaxially with dark, triangular areas in axils of two basal secondary veins, with dense, glandular dots dark purple or green; leaves of sprouting branches heteromorphic, blades nearly orbiculate, 3–5-lobed, lobes cuspidate or caudate at the apex, abaxially with 3–4 nectaries at the axils of lower secondary veins. **Inflorescences**: racemose-corymbose, terminal, 3–13-flowered, rarely partly branched, with peduncles 2–2.5 cm long, scattered narrowly elliptic lenticels, glabrous, with bracts at the base of each pedicel; pedicel 2–3.5 cm long, with 1–3 bracteoles below the middle of each pedicel, bracteoles alternate, shorter than bracts, narrowly linear, 2–6 × 0.4–1 mm, curved at the apex, glabrous, deciduous after anthesis. **Calyx**: turbinate, red-purple, glabrous, 2-parted, near ca. 1 mm from the base, lobes ovate to broadly elliptic, 1.2–1.8 × 0.6–0.9 cm, cuspidate at the apex, tipped with two abrupt points. **Corolla**: tubular–funnelform, 3.5–4.2 cm long, glabrous, tube 1.8–2.1 cm long, ca. 2 mm wide at base, 1.6–1.9 cm wide at orifice of corolla, transversely elliptic, occasionally with simple hairs inside the throat. limb 2-lipped, lower lip 3-lobed, lobes transversely broadly elliptic to orbiculate, 1.4–1.8 × 1.9–2.2 cm, rounded or obtuse at the apex, connate at the base for 5–9 mm; upper lip 2-lobed, lobes broadly ovate to transversely oblong, 0.5–0.7 × 1.1–1.3 cm, rounded or obtuse at the apex, connate at the base for 0.9–1.2 cm, all lobes crimped; corolla white, with many small purple spots on the tube outside; lower lobes with rows of larger purple spots along the veins and densely scattered over the surface and inside of the tube, including some spots exceeding 1 mm in diameter; with two rows of disjunct bright yellow spots and slightly raised 2-longitudinal ridges between the tube inside and the lower lobes. Stamens 2, 1.3–1.6 cm long, glabrous; filaments: white, scattered purple dots; anthers: yellow, two anther cells divaricate, ovate-elliptic, 2.5–3 mm long; staminodes 3, ca. 4 mm long, white, glabrous, stamens and staminodes inserted into the corolla tube ca. 2 mm from the base. Pistil glabrous; ovary 2–3 mm long, green; style 1.4–1.7 cm long, white; stigma 2-parted, lobes lanceolate unequal, 1.5–2 mm long, purplish. **Capsules**: 1–2 per infructescence, 30–70 cm long, 3–5 mm wide, glabrous, with the septum 1.5–2.5 mm wide. **Seeds**: body brown, transversely narrowly oblong, 1.5–2 mm long, 0.9–1.5 cm wide, truncate at the base and apex, sides acute, ciliate on sides, with hairs 0.5–2 cm long, white.

##### Distribution.

Wild populations of *C. lutea* are found in Shaanxi, Henan, Shandong, Anhui, and Jiangsu provinces, with relatively abundant populations preserved in mountainous areas of coastal regions. Natural populations are distributed at elevations of approximately 0–800 m a.s.l. The species is currently widely cultivated in northern China.

##### Phenology.

Flowering occurs from mid-April to early May, lasting 10–15 days, and fruiting extends from May to September. The flowering period begins approximately 8 days earlier than that of sympatrically distributed *C. bungei*.

##### Etymology.

The specific epithet of ‘lutea’ comes from local Chinese names: “Huang Qiu” (Yellow *Catalpa*, in Henan Province, China) and “Jin Qiu” (Golden *Catalpa*, in Shandong Province, China) refer to yellow heartwood.

##### Habitat and ecology.

Through a two-year investigation of wild *Catalpa* resources across more than 40 prefectures and cities in China, we documented the habitat and ecological characteristics of *C. lutea*. It is distributed in the temperate regions of China, with its range partially overlapping those of *C. bungei* and *C. fargesii* (Fig. 1). However, it is the sole *Catalpa* species documented in the eastern coastal region. For instance, it can be found in the granitic rocky habitats of Yuntai Mountain in Lianyungang, at elevations of 200–450 m a.s.l., where the environment is dominated by weathered scree slopes with only thin layers of acidic humus soil retained in rock crevices. Associated species include *Selaginella sinensis* and scattered *Ulmus parvifolia*. The new species roots directly in rock fissures, demonstrating lithophytic adaptation. In other distribution areas, it grows on slopes and in forests, accompanied by trees such as *Cercidiphyllum japonicum* Sieb. et Zucc., *Toona sinensis* (Juss.) Roem., *Diospyros kaki* var. *silvestris* Makino, *Chionanthus retusus* Lindl. & Paxton, and *Cornus officinalis* Sieb. et Zucc.; shrubs such as *Salix caprea* L. and *Spiraea chinensis* Maxim.; as well as herbaceous plants including *Teucrium viscidum* Bl., *Geranium wilfordii* Maxim., *Impatiens pterosepala* Hook. f., *Silene tatarinowii* Regel, *Artemisia lavandulifolia* DC., and *Artemisia sacrorum* Ledeb., among others. Unlike *C. duclouxii*, *C. lutea* produces relatively fewer fruits. In certain habitats, it relies primarily on sprouting propagation, while in others it does set fruit.

## Discussion

*Catalpa* has a series of distinguishable characteristics, such as linear capsules, spindle-shaped seeds, and a campanulate corolla. *Catalpa* with yellow heartwood has been recorded from China around 544 CE, but the taxonomic status of this taxon has remained uncertain. Through a nationwide survey of wild *Catalpa* resources across China, together with comparative analyses of morphology, distribution, and phenology, as well as phylogenetic reconstructions based on genome-wide SNPs and complete chloroplast genomes, we confirm that this lineage is clearly distinct from other species in sect. *Catalpa*. In accordance with its diagnostic yellow anthers and heartwood, we formally describe it here as *C. lutea*.

Among the Chinese *Catalpa* species, *C. ovata* can be readily distinguished from other native taxa listed in Table 1 by its paniculate inflorescences bearing 30–240 flowers, pale yellow to white corolla base, and infructescences producing 20–35 capsules. However, the taxonomic treatment of the remaining Chinese lineages has been subject to considerable controversy.

Taking *C. duclouxii* as an example, Dode (1907) described this species based on specimens from Yunnan, noting its glabrous leaf undersurfaces and inflorescences, along with more highly branched inflorescences, as key differences from *C. fargesii*. Rehder (1913) later treated it as a variety of *C. fargesii*, and Gilmour (1936) further reduced its status to a form of *C. fargesii*. However, Li (2008) revealed that *C. duclouxii* is genetically closer to *C. bungei* and argued against its classification as an infraspecific taxon of *C. fargesii*. Olsen and Kirkbride (2017), in contrast, considered that the three taxa—*C. bungei* (with simple hairs), *C. fargesii* (with stellate pubescence), and *C. duclouxii* (glabrous)—could not be reliably distinguished based solely on indumentum characteristics and thus merged them into a single species.

Our long-term observations reveal that *C. duclouxii* exhibits a narrower crown compared to *C. bungei* and *C. fargesii*. Its inflorescences are racemose to paniculate, bearing (12–)15–38 or more flowers, and it sets a higher number of fruits (Table 1). Pollen viability follows the order *C. duclouxii* > *C. bungei* > *C. fargesii* (Jia 2009). This species is widely distributed in southwestern China across an elevational range of 400–2800 m a.s.l., representing the highest altitudinal distribution among the native taxa. Its range only partially overlaps with those of *C. bungei* and *C. fargesii*, and its phenology differs significantly. In our phylogenetic reconstructions, samples of *C. duclouxii* consistently form a monophyletic clade. The phylogeny based on genome-wide SNPs places it closer to *C. bungei*, whereas the chloroplast genome tree shows a conflicting topology—a pattern congruent with findings from Li (2008) and Dong et al. (2022). This suggests potential incomplete lineage sorting or introgression among *C. bungei*, *C. fargesii*, and *C. duclouxii*. Nevertheless, the pronounced morphological, ecological, phenological, and adaptive divergences among them support their recognition as distinct species.

Regarding *C. fargesii*, Bureau (1894) accepted it as a valid species in the first comprehensive monograph of *Catalpa*. Although Olsen and Kirkbride (2017) proposed its merger with *C. bungei* and *C. duclouxii*, our study identifies several distinguishing morphological traits (Table 1). Furthermore, *C. fargesii* is the only one of the three that extends into relatively higher latitudes. Therefore, we maintain that these three lineages should be treated as separate species.

The new species described herein is morphologically more distinct from the above three lineages (Figs 4, 5; Table 1). Ma et al. (2020), using AFLP markers to compare 75 samples of ‘Jinsiqiu’ (the provisional name for *C. lutea*) with *C. bungei* and *C. fargesii*, reported significant morphological and genetic differentiation, with a considerable genetic distance, and recommended a revision of its taxonomic status. Li et al. (2022) also explored its phylogenetic relationships using chloroplast genome data. Through nationwide field surveys, we have identified a set of consistent diagnostic characters and a distinct distribution range for this taxon. Moreover, in areas where its range overlaps with *C. bungei*, it flowers approximately 8 days earlier, indicating a degree of reproductive isolation.

*C. lutea* primarily propagates through sprouting and rarely sets fruit. Its capsules measure approximately 40–70 cm in length, with fusiform seeds bearing white hairs at both ends. The corolla is 5-lobed, featuring obovate lobes. Wild populations of this species are documented in Henan, Shaanxi, Hebei, Shandong, Anhui, and Jiangsu provinces, with relatively well-preserved populations found in coastal mountainous areas. Compared to other species in the genus *Catalpa*, *C. lutea* exhibits distinctive characteristics such as distinctly yellow heartwood, bright yellow anthers, filaments with purple dots, and darker, thicker, and more prominent deep purple stripes inside the corolla tube, along with floral dots exceeding 1 mm in diameter.

### Specimens examined

**China • Henan**: Luanchuan County, Qiuba Township, 34°07'59.6"N, 111°30'30.96"E, 115 m a.s.l., on slopes, 23 April 2020, JT Zhang, JSQ-2020-001 (PE and CAF), JSQ-2020-002 (PE and CAF), and JSQ-2020-003 (PE and CAF); • **Jiangsu**: Lianyungang City, Haizhou District, Huaguoshan, 34°39'2.47"N, 119°17'14.03"E, 495 m a.s.l., on slopes, 23 April 2023, Y Liu, JSQ-2023-003 (CAF); • **Shandong**: Linyi City, Mengyin County, Xinzhongshan Village, 35°48'38.26"N, 118°12'51.84"E, 314 m a.s.l., 05 May 2023, Y Liu, JSQ-2023-010 (CAF).

### Proposed IUCN conservation status

The species is considered to be “Data Deficient” (DD) according to the IUCN Red List Categories and Criteria (IUCN 2017).

## Supplementary Material

XML Treatment for Catalpa lutea

## Additional information

### Use of AI

No use of AI was reported.

### Author ORCIDs

Ying Liu https://orcid.org/0000-0002-2905-0015

Zhen-Yu Li https://orcid.org/0009-0007-1224-0692

Jiang-Tao Zhang https://orcid.org/0009-0007-6417-7015

Wan-Ting Ge https://orcid.org/0009-0005-4009-8481

Ming-Gang Zhang https://orcid.org/0009-0005-2914-4360

Nan Lu https://orcid.org/0000-0001-7736-5217

Wen-Jun Ma https://orcid.org/0000-0002-1956-3765

Jun-Hui Wang https://orcid.org/0000-0003-0384-4746

### Data availability

All of the data that support the findings of this study are available in the main text or Supplementary Information.

## Supplementary materials

Supplementary material 1 Materials used in the present study Data type docx Explanation note All samples were used for whole-genome resequencing and chloroplast genome assembly. https://binary.pensoft.net/file/1522262This dataset is made available under the Open Database License (http://opendatacommons.org/licenses/odbl/1.0/). The Open Database License (ODbL) is a license agreement intended to allow users to freely share, modify, and use this Dataset while maintaining this same freedom for others, provided that the original source and author(s) are credited. Ying Liu, Zhen-Yu Li, Jiang-Tao Zhang, Wan-Ting Ge, Ming-Gang Zhang, Nan Lu, Wen-Jun Ma, Jun-Hui Wang
