# Supplementary material for: Catalpa lutea (Bignoniaceae), a new species from north, central, and east China
Source: PhytoKeys. 2026 Jan 28;270:25–39. doi: 10.3897/phytokeys.270.171460 (PMC12873570; doi:10.3897/phytokeys.270.171460)
Supplement: Supplementary material 1 — Materials used in the present study [file phytokeys-270-025_article-171460__-s001.docx]

**Table S1.** Materials used in the present study. All samples were used for whole-genome resequencing and chloroplast genome assembly.

| **Accession number** | **Species** | **Collection locality** | **Preservation** | **DNA source** |
| --- | --- | --- | --- | --- |
| BOP5 (MW1) | *Chilopsis linearis* | Yavapai County, Arizona, USA | Herbarium | leaf |
| Big02 | *Catalpa bignonioides* | Introduced in Gansu (originally from U.S. National Arboretum, Washington, DC, USA) | Silicagel | leaf |
| Big08 | *Catalpa bignonioides* | Introduced in Gansu (originally from U.S. National Arboretum, Washington, DC, USA) | Silicagel | leaf |
| SP2 | *[Catalpa speciosa](https://www.iplant.cn/info/Catalpa speciosa" \o "https://www.iplant.cn/info/Catalpa speciosa)* | Introduced in Gansu (originally from U.S. National Arboretum, Washington, DC, USA) | Silicagel | leaf |
| SP3 | *[Catalpa speciosa](https://www.iplant.cn/info/Catalpa speciosa" \o "https://www.iplant.cn/info/Catalpa speciosa)* | Beijing, China | Silicagel | leaf |
| SP4 | *[Catalpa speciosa](https://www.iplant.cn/info/Catalpa speciosa" \o "https://www.iplant.cn/info/Catalpa speciosa)* | Beijing, China | Silicagel | leaf |
| zsy2 | *Catalpa ovata* | Hehua Village, County Road, Duyun City, Guizhou Province, China | Silicagel | leaf |
| zsy4 | *Catalpa ovata* | Ganxi Village, Enshi City, Enshi Tujia and Miao Autonomous Prefecture, Hubei Province, China | Silicagel | leaf |
| zsy5 | *Catalpa ovata* | Banbian Street, Guiding County, Guiyang City, Guizhou Province, China | Silicagel | leaf |
| dqy5 | *Catalpa duclouxii* | Nanying Village, Heqing County, Dali Prefecture, Yunnan Province, China | Silicagel | leaf |
| dqy6 | *Catalpa duclouxii* | Mizong Village, Dali Bai Autonomous Prefecture, Yunnan Province, China | Silicagel | leaf |
| dqy8 | *Catalpa duclouxii* | Majia Yi Village, Shangguan Town, Dali Bai Autonomous Prefecture, Yunnan Province, China | Silicagel | leaf |
| dqy33 | *Catalpa duclouxii* | Huating Temple, Xishan, Kunming City, Yunnan Province, China | Silicagel | leaf |
| dqy7 | *Catalpa duclouxii* | Guanying Village, Eryuan County, Dali Bai Autonomous Prefecture, Yunnan Province, China | Silicagel | leaf |
| dqy35 | *Catalpa duclouxii* | Majia Yi Village, Shangguan Town, Dali Bai Autonomous Prefecture, Yunnan Province, China | Silicagel | leaf |
| hq025 | *[Catalpa fargesii](https://www.iplant.cn/info/Catalpa fargesii" \o "https://www.iplant.cn/info/Catalpa fargesii)* | Pingliang City, Gansu Province, China | Silicagel | leaf |
| hqy16 | *[Catalpa fargesii](https://www.iplant.cn/info/Catalpa fargesii" \o "https://www.iplant.cn/info/Catalpa fargesii)* | Qingyang City, Gansu Province, China | Silicagel | leaf |
| hqy49 | *[Catalpa fargesii](https://www.iplant.cn/info/Catalpa fargesii" \o "https://www.iplant.cn/info/Catalpa fargesii)* | Xianyang City, Shaanxi Province, China | Silicagel | leaf |
| jsy32 | *Catalpa lutea* | Linyi City, Shandong Province, China | Silicagel | leaf |
| jsy41 | *Catalpa lutea* | Weifang City, Shandong Province, China | Silicagel | leaf |
| jsy45 | *Catalpa lutea* | Haoshan Village, Shiqiao Town, Qiyuan County, Zibo City, Shandong Province, China | Silicagel | leaf |
| js5 | *Catalpa lutea* | Huaguoshan, Haizhou District, Lianyungang City, Jiangsu Province, China | Silicagel | leaf |
| js12 | *Catalpa lutea* | Wanshou Valley, Beiyuntai Mountain, Lianyun District, Lianyungang City, Jiangsu Province, China | Silicagel | leaf |
| js22 | *Catalpa lutea* | Xinzhongshan Village, Tanbu Town, Mengyin County, Linyi City, Shandong Province, China | Silicagel | leaf |
| jsy31 | *Catalpa lutea* | Linyi City, Shandong Province, China | Silicagel | leaf |
| qs5 | *[Catalpa bungei](https://www.iplant.cn/info/Catalpa bungei" \o "https://www.iplant.cn/info/Catalpa bungei)* | Yu'an District, Liu'an City, Anhui Province, China | Silicagel | leaf |
| qs9 | *[Catalpa bungei](https://www.iplant.cn/info/Catalpa bungei" \o "https://www.iplant.cn/info/Catalpa bungei)* | Zhenglou Village, Chaiji Town, Funan County, Anhui Province, China | Silicagel | leaf |
| qs20 | *[Catalpa bungei](https://www.iplant.cn/info/Catalpa bungei" \o "https://www.iplant.cn/info/Catalpa bungei)* | Yanzhuang Village, Tanpeng Town, Linquan County, Fuyang City, Anhui Province, China | Silicagel | leaf |
| qs11 | *[Catalpa bungei](https://www.iplant.cn/info/Catalpa bungei" \o "https://www.iplant.cn/info/Catalpa bungei)* | Liuluya Village, Xincun Town, Funan County, Fuyang City, Anhui Province, China | Silicagel | leaf |
| qs14 | *[Catalpa bungei](https://www.iplant.cn/info/Catalpa bungei" \o "https://www.iplant.cn/info/Catalpa bungei)* | Within the bamboo forest, Xiaotaizhuang Village, Funan County, Fuyang City, Anhui Province, China | Silicagel | leaf |
